# Deficiency of Gankyrin in the small intestine is associated with augmented colitis accompanied by altered bacterial composition of intestinal microbiota

**DOI:** 10.1186/s12876-019-1156-0

**Published:** 2020-01-15

**Authors:** Toshiharu Sakurai, Hiroki Nishiyama, Tomoyuki Nagai, Susumu Goto, Hiroyuki Ogata, Masatoshi Kudo

**Affiliations:** 1 Department of Gastroenterology and Hepatology, Kindai University Faculty of Medicine, 377-2, Osaka-Sayama, Osaka 589-8511 Japan; 2 Institute for Chemical Research, Kyoto University, Uji, 611-0011 Japan; 3 Database Center for Life Science, Joint-Support Center for Data Science Research, Research Organization of Information and Systems, Wakashiba, Kashiwa, Chiba 277-0871 Japan

**Keywords:** Inflammatory bowel disease, Gankyrin, Microbiota, *Helicobacter japonicum*, *Bilophila*

## Abstract

**Background:**

Gankyrin (GK) is an oncoprotein which regulates inflammatory responses and its inhibition is considered as a possible anti-inflammatory therapy for inflammatory bowel disease (IBD).

**Methods:**

In this study, we investigated the role of GK in epithelial cells using mice with intestinal epithelial cell-specific GK deletion in (i) the entire small intestine and colon (*Villin-Cre;Gankyrin*^*f/f*^) and (ii) the distal intestine and colon (*Cdx2-Cre;Gankyrin*^*f/f*^).

**Result:**

Unexpectedly, GK-deficiency in the upper small bowel augmented inflammatory activity compared with control mice when colitis was induced with dextran sodium sulfate. Biochemical analyses have revealed GK-deficiency to have caused reduction in the expression of antimicrobial peptides, α-Defensin-5 and -6, in the upper small bowel. Examination of human samples have further confirmed that the reduction of GK expression in the small bowel is associated with colonic involvement in human Crohn’s disease. Through the sequencing of bacterial 16S rRNA gene amplicons, bacteria potentially deleterious to intestinal homeostasis such as *Helicobacter japonicum* and *Bilophila* were found to be over-represented in colitis induced *Villin-Cre;Gankyrin*^*f/f*^ mice when compared to *Gankyrin*^*f/f*^ control mice under the same condition.

**Conclusion:**

These results highlight the distinct site dependence of the pro- and anti-inflammatory functions of GK and provide important insights into the pathogenesis of IBD.

## Background

Inflammatory bowel disease (IBD) is characterized by a persistent inflammation in the colon and/or small intestine. Crohn’s disease (CD) may affect any segment of the digestive tract, while ulcerative colitis is restricted to the colon. IBD arises due to disruption of immune tolerance to the gut microbiota that leads to chronic intestinal inflammation and mucosal damage in genetically predisposed hosts [1, 2]. The gut microbiota is known to be altered in patients with IBD and contributes to the pathogenesis of IBD. The incidence of IBD is increasing worldwide owing to the socioeconomic development and the environmental factors associated with modern life, that appear to promote the changes in gut microbiota [1, 2].

In IBD, long-standing inflammation is a major risk factor for colorectal cancer, also called as colitis-associated cancer (CAC) [3]. Indeed, patients with refractory IBD exhibit a higher risk of colorectal cancer than individuals in the general population [4]. In our previous reports, we found that chronic inflammation induces stress response proteins and Gankyrin (GK) (also known as PSMD10, p28 and Nas6p), which promote the development of CAC [5–8]. A significant correlation was found between the expression of these molecules and stem cell markers in the colonic mucosa of IBD patients. In addition, stress response protein heat shock protein A4 (HSPA4) and stem cell marker, B cell-specific Moloney murine leukemia virus insertion site 1 (Bmi1), could predict poor therapeutic response to steroid and anti-TNF-α antibodies in IBD patients [6, 9]. GK upregulates the expression of pro-inflammatory cytokines (TNF-α and IL-17) and stem cell markers, Bmi1 and sex-determining region Y (SRY)-box 9 (Sox9), in the colon. These data suggest that persistent inflammation can result in resistance to treatment and refractory clinical course in patients with IBD and eventually increases the risk of CAC through the major role played by GK.

To examine whether inhibition of GK might prevent colitis, we have employed 2 kinds of epithelial cell-specific GK-deficient mice, i.e. (i) small intestine and colon-specific GK-deficient mice (*Villin-Cre;Gankyrin*^*f/f*^) and (ii) distal intestine and colon-specific GK-deficient mice (*Cdx2-Cre;Gankyrin*^*f/f*^). These mice were challenged by dextran sodium sulfate (DSS) which is a colitis-inducing agent. Surprisingly, *Villin-Cre;Gankyrin*^*f/f*^ mice were more susceptible to DSS-induced colitis and showed an alteration of gut microbiota in comparison with *Gankyrin*^*f/f*^ control mice. In contrast, *Cdx2-Cre;Gankyrin*^*f/f*^ showed a similar level of inflammatory response as *Gankyrin*^*f/f*^ control mice. This study suggests that GK in the upper small bowel is involved in the pathogenesis of colitis through affecting gut microbiota.

## Methods

### Knock out mice and treatment

*Gankyrin*^*flox/flox*^ [8, 10], *Villin-Cre* and *Cdx2-Cre* mice (obtained from Jackson laboratory) were used to produce tissue-specific conditional GK knockout mice, designated as *Villin-Cre;Gankyrin*^*f/f*^ and *Cdx2-Cre;Gankyrin*^*f/f*^ mice, respectively. All mice were maintained in a specific-pathogen-free environment and housed under a 12-h dark-light cycle (light from 7:00 to 19:00). They were given free access to standard diet and water and were not fasted before the experiments. Sex- and age-matched *Cdx2-Cre;Gankyrin*^*f/f*^, *Villin-Cre;Gankyrin*^*f/f*^ [8], and *Gankyrin*^*f/f*^ (control) mice (8–16 weeks old) were administered with 2.5% (w/v) dextran sodium sulfate (DSS; molecular weight, 36,000–50,000 kDa; MP Biomedicals, Solon, OH) in drinking water for 7 days. Inflammatory cell infiltration score was assessed using a method described in a previous study (*Villin-Cre;Gankyrin*^*f/f*^ mice *n* = 6, *Gankyrin*^*f/f*^ mice *n* = 5) [5]. The pathological findings were blindly examined by TS and TN. Mice were sacrificed by cervical dislocation under deep isoflurane anesthesia. After the mice were sacrificed, the colon was excised from the ileocecal junction to the anus and the sections were longitudinally cut to prepare them for histological evaluation.

All animal procedures were performed according to approved protocols and in accordance with the recommendations for the proper care and use of laboratory animals. The animal study protocol was approved by the Medical Ethics Committee of Kindai University Faculty of Medicine and Institutional Animal Care (#23–098, #28–224).

### Human tissue samples

The upper small intestine tissues (*n* = 20) were obtained by endoscopy from patients with CD. Informed consent of all the patients was obtained. The clinical study protocol conformed to the ethical guidelines of the 1975 Declaration of Helsinki and was approved by the institutional review board of Kindai University Faculty of Medicine.

### Biochemical analyses

Real-time qPCR and immunohistochemistry were performed as previously described in the respective references [7, 11, 12]. The primer sequences for mouse tumor necrosis factor (TNF)-α, tumor growth factor (TGF)-β, R-spondin, wingless-type MMTV integration site 5B (Wnt5B), α-Defensin 5 (Defa5), α-Defensin 6 (Defa6), interferon (IFN)-α, and IFN-γ were: 5′-GACCAGGCTGTCGCTACATCA-3′ and 5′-CGTAGGCGATTACAGTCACGG-3′; 5′-GACTCTCCACCTGCAAGACC-3′ and 5′-GACTGGCGAGCCTTAGTTTG-3′; 5′-ATTCTGCTGGAGAGGAACGA-3′ and 5′-GTGCTCGATCTTGCATTTGA-3′; 5′-CCGAGAGCGTGAGAAGAACT-3′, and 5′-GGCGACATCAGCCATCTTAT-3′; 5′-TATCTCCTTTGGAGGCCAAG-3′ and 5′-TTTCTGCAGGTCCCAAAAAC-3′; 5′-GTCCAGGCTGATCCTATCCA-3′ and 5′-GACACAGCCTGGTCCTCTTC-3′; 5′-AGTGAGCTGACCCAGCAGAT-3′ and 5′-GGTGGAGGTCATTGCAGAAT-3′; 5′-ACTGGCAAAAGGATGGTGAC-3′ and 5′-GCTGATGGCCTGATTGTCTT-3′. The upper small bowel tissues from colitis non-induced *Villin-Cre;Gankyrin*^*f/f*^ mice and their corresponding floxed mice were collected and total RNA was extracted using RNeasy kit (QIAGEN, Hilden, Germany). Microarray analysis was performed by Hokkaido System Science Co., Ltd. (Sapporo, Japan).

### DNA extraction and sequencing

Frozen samples of caecum and rectum were thawed and homogenized through the usage of Zirconia/Silica Beads (BioSpec Products) and MagNALyzer (Roche Diagnostics). Next, DNA was extracted from the homogenized samples by the usage of QIAamp DNA Mini Kit according to the manufacturer’s instructions (Qiagen GmbH, Hilden Germany). The variable V3–V4 16S rRNA gene regions of the extracted DNA samples were amplified by PCR with 16S Amplicon PCR Forward primer 5′-TCGTCGGCAGCGTCAGATGTGTATAAGAGACAG -MID-GT-CCTACGGGNGGCWGCAG-3′ and 16S Amplicon PCR Reverse primer 5′-GTCTCGTGGGCTCGGAGATGTGTATAAGAGACAG-MID-GT-GACTACHVGGGTATCTAATCC-3′. The preparation of sequencing libraries was conducted according to the protocol described in ‘16S Metagenomic Sequencing Library Preparation: Preparing 16S Ribosomal RNA Gene Amplicons for the Illumina MiSeq System’ protocol [13] with the usage of the Nextera XT Index Kit (Illumina). The MiSeq Reagent Kit v2 (300 cycles) and MiSeq (Illumina, San Diego, CA, USA) device was used for the sequencing of the samples.

### Bioinformatics analysis of 16S rRNA amplicon sequences

Amplicon sequences were processed with the following procedures modified from our previous paper [14]. Low-quality and primer regions were removed from each paired-end reads using Trimmomatic (version 0.35) (PE, SLIDINGWINDOW:40:15, MINLEN:50) [15] and Cutadapt (version 1.11) (-e 0.18, --pair-filter = both) [16], respectively. Paired-end reads were aligned to make merged reads (i.e. “reads” hereafter) using FLASH (version 1.2.11) (-m 30, -M 271, -x 0.25) [17].

The representative 16S rRNA sequences of Greengenes database (version 13_8, 99_otus.fasta) [18] were used to cluster reads into OTUs at 99% identity using UCLUST [19] implemented in Quantitative Insights Into Microbial Ecology’s (QIIME version 1.9.1) [20] parallel_pick_otus_uclust_ref.py (--max_rejects 0, --enable_rev_strand_match). Taxonomic groups were assigned to OTUs based on ‘99_otu_taxonomy.txt’ in Greengenes database. Reads that were not assigned to OTUs at this stage were checked for chimera using uchime algorithms [21] implemented in VSEARCH (version 2.10.4), (--uchime_denovo and --uchime_ref, --db 99_otus.fasta) [22]. Identified chimeric sequences were discarded. Remaining unclassified reads were grouped into additional OTUs at 99% identity using USEARCH [19] implemented in QIIME’s pick_otus.py (--otu_picking_method usearch61, --similarity 0.99, --enable_rev_strand_match). Of these OTUs, singletons and doubletons were discarded. Taxonomy was assigned to the most abundant sequence of each OTU using the RDP classifier [23] in QIIME’s parallel_assign_taxonomy_rdp.py (--confidence 0.8, --id_to_taxonomy_fp 99_otu_taxonomy.txt, --reference_seqs_fp 99_otus.fasta) with training on Greengenes database.

Rarefaction curves were generated by randomly sampling reads from each sample 10 times and calculating the average number of OTUs across different sampling depths with intervals of 5000 reads using QIIME’s parallel_multiple_rarefactions.py. OTU richness and Shannon diversity index [24] values were calculated based on reads randomly sampled at the depth of the smallest sample size with QIIME’s alpha_rarefaction.py. This was repeated 10 times for each sample and their average was used for the non-parametric two-sample *t*-test implemented in QIIME’s compare_alpha_diversity.py with 10,000 Monte Carlo permutations. The *P*-values were adjusted for multiple testing using Holm’s method.

Beta diversity analysis was performed using unweighted and weighted UniFrac [25] distances after randomly sub-sampling from each sample using QIIME’s beta_diveristy.py. The sampling depth was set as the read count of the smallest sample. The phylogenetic tree used for the measurement of UniFrac distance was created based on the representative reads (i.e. the most abundant read in each OTU) using SSU-ALIGN (version 0.1.1) [26] and FastTree [27] in QIIME’s make_phylogeny.py. Principal coordinate analysis was conducted on the UniFrac distances using QIIME’s principal_coordinates.py.

The significance of the compositional difference between two groups of microbiotas was tested using Adonis [28] implemented in QIIME’s compare_categories.py with 10,000 permutations. When the number of samples was not large enough to conduct 10,000 permutations, the maximum permutation size was automatically selected by the program. To compensate for UniFrac distance’s sensitivity to subsampling, the process of subsampling, measuring UniFrac distances and calculating Adonis *P*-value was repeated 10 times. We used the average of the *P*-values. The *P*-values were adjusted for multiple testing using Holm’s method.

Prior to testing the significance of differential abundance of OTUs, OTUs supported by less than a total of 100 reads from samples in the pair of groups under comparison were omitted from analysis. Furthermore, OTUs observed in less than 25% of the total number of samples under comparison were also removed. These steps were done using QIIME’s filter_otus_from_otu_table.py. The statistical significance of the differential abundance of OTUs between sample groups were tested using the DESeq2’s negative binomial Wald test [29] implemented in QIIME’s differential_abundance.py. False discovery rate was calculated with the Benjamini-Hochberg correction and the cut-off was set at 0.05. For each OTU that showed significant differential abundance, the sample group with the higher average relative abundance was examined. If any of the samples belonging to this group had no reads for the OTU in question, we did not consider the OTU to be differentially represented. Furthermore, if the maximum relative abundance of the OTU in the low-abundance group was higher than the relative abundance of the same OTU in more than half of the samples of the high-abundance group, the OTU was not considered to be differentially represented.

In some specific cases, we inspected detailed taxonomy of OTUs using homology searches. Homology searches of the representative sequences of OTUs (i.e., full length Greengenes sequences or the most abundant read if former is not available) against the NCBI’s nucleotide collection (nr/nt) database were conducted using the online megablast tool [30, 31] with the default settings.

### Statistical analysis

Statistical tests were performed using two-tailed Student’s *t*-test with *P* value < 0.05 as the significance cut-off, unless otherwise stated.

## Results

### Mice that lack GK levels in epithelial cells in the upper small intestine are hypersusceptible to DSS-induced colitis

**Fig. 1:**
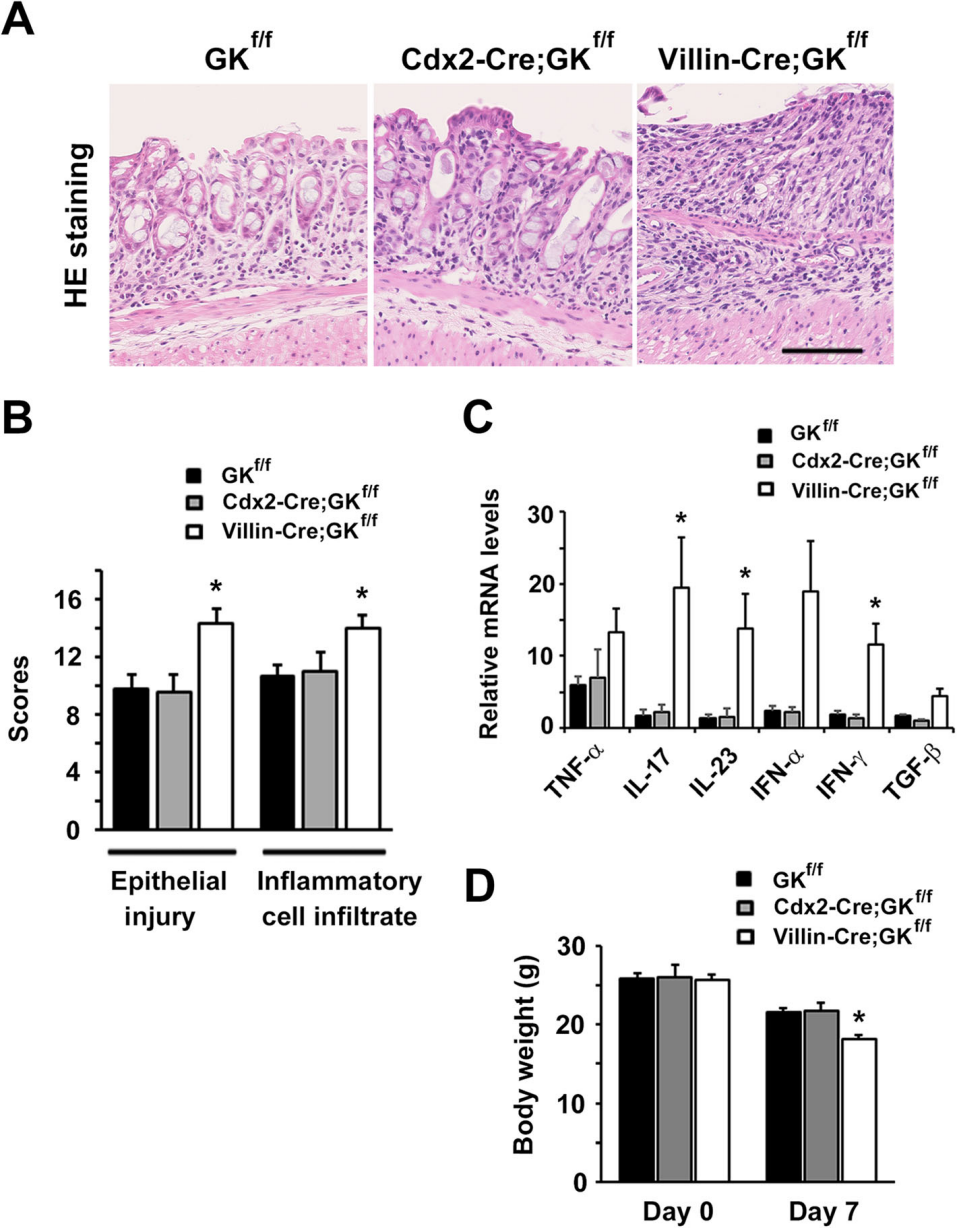
Mice that lack Gankyrin (GK) levels in epithelial cells in the upper small intestine are hypersusceptible to dextran sodium sulfate (DSS)-induced colitis. (**A**) Representative images of hematoxylin and eosin stained colonic tissues in *Gankyrin*^*f/f*^ (GK^f/f^), *Cdx2-Cre;Gankyrin*^*f/f*^ (Cdx2-Cre;GK^f/f^) and *Villin-Cre;Gankyrin*^*f/f*^ (Villin-Cre;GK^f/f^) mice. Cdx2-Cre;GK^f/f^, Villin-Cre;GK^f/f^ and GK^f/f^ mice were administered with 2.5% DSS in drinking water for 7 days. Scale bar, 100 μm. (**B**) Epithelial injury and inflammatory cell infiltration into colonic tissues of GK^f/f^, Cdx2-Cre;GK^f/f^, and Villin-Cre;GK^f/f^ mice were observed after 7 days of the initiation of DSS administration. *n* = 6 per group. (**C**) RNA was extracted from colonic tissues of DSS-treated Villin-Cre;GK^f/f^ (n = 6), Cdx2-Cre;GK^f/f^ (*n* = 4), and GK^f/f^ (*n* = 5) mice. Relative quantities of mRNA were determined by real-time qPCR and were normalized to the quantity of β-actin mRNA. The relative quantity of each mRNA in the untreated colon was assumed to be an arbitrary value of 1.0. Data are represented as mean ± SEM. (**D**) Body weight of DSS-treated Villin-Cre;GK^f/f^ (n = 6), Cdx2-Cre;GK^f/f^ (n = 4), and GK^f/f^ (n = 5) mice were shown at the time point of day 0 and day 7

Initially we tried to determine the role of GK in the development of inflammation in the colon. To achieve this, we produced *Gankyrin*^*f/f*^ mice [8] and crossed them with (i) *Villin-Cre* and (ii) *Cdx2-Cre* transgenic mice to generate mice that lack GK levels in their intestinal epithelial cells of (i) the whole small intestine and the colon (*Villin-Cre;Gankyrin*^*f/f*^ mice) and (ii) the distal small intestine and the colon (*Cdx2-Cre;Gankyrin*^*f/f*^ mice), respectively (Additional file 1: Figure S1). Experimental colitis was induced by treating the mice with 2.5% DSS for 7 days. The degree of inflammatory cell infiltration into the colon and the epithelial injury were higher in *Villin-Cre;Gankyrin*^*f/f*^ mice (*n* = 6) than in *Gankyrin*^*f/f*^ control mice (*n* = 5) (*P* < 0.05, Fig. 1a and b). Furthermore, we investigated the change in immune response associated with GK-deficiency by analyzing the cytokine levels in the colon. Colonic tissues from *Villin-Cre;Gankyrin*^*f/f*^ mice showed an enhanced immune response with high levels of pro-inflammatory cytokines IL-17A, IL-23, and IFN-γ (Fig. 1c). In the DSS-induced colitis model, body weight of mice reflects the extent of colonic inflammation [6]. On day 7, a significant difference was seen in body weight between *Villin-Cre;Gankyrin*^*f/f*^ and control mice treated with DSS. In contrast, *Cdx2-Cre;Gankyrin*^*f/f*^ and control mice showed similar levels of body weight (Fig. 1d). The GK-deficiency in epithelial cells of the upper small bowel, but not in the colon, had augmented the colonic inflammation.

### The reduced expression of antimicrobial peptides in GK-deficient intestinal epithelium

**Fig. 2:**
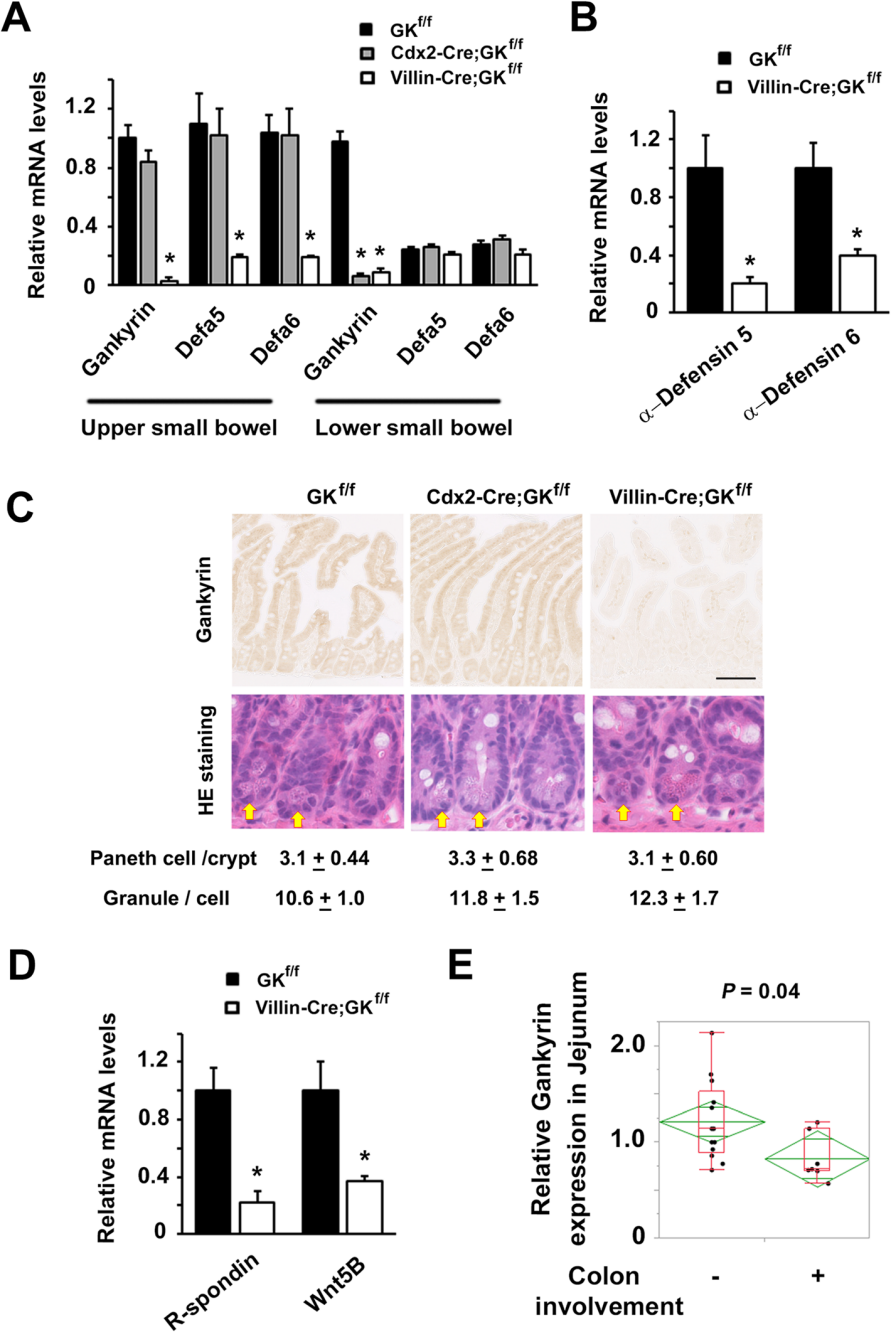
The reduced expression of antimicrobial peptides in GK-deficient intestinal epithelium. RNA was extracted from (**A**) the upper small bowel and the lower small bowel tissues of untreated *Villin-Cre;Gankyrin*^*f/f*^ (Villin-Cre;GK^f/f^) and *Gankyrin*^*f/f*^ (GK^f/f^) mice, and (**B**) the upper small bowel tissues of DSS-treated Villin-Cre;GK^f/f^ and GK^f/f^ mice. The relative quantities of mRNA in all the above samples were determined by real-time qPCR and were normalized to the quantity of β-actin mRNA. The relative quantity of each mRNA in the untreated small intestine samples was assumed to be an arbitrary value of 1.0. Data are represented as mean ± SEM (*n* = 3). (**C**) Immunohistochemistry using anti-GK antibody and Hematoxylin & Eosin (HE) staining were performed on the upper small bowel samples of Villin-Cre;GK^f/f^, Cdx2-Cre;GK^f/f^, and GK^f/f^ mice at day 7. Arrows indicate Paneth cells at the base of the intestinal glands. The numbers of Paneth cells per crypt and granules per one Paneth cell were shown. (**D**) RNA was extracted from the upper small bowel tissues of untreated Villin-Cre;GK^f/f^ and GK^f/f^ mice. The relative quantities of mRNA in all the above samples were determined by real-time qPCR and were normalized to the quantity of β-actin mRNA. The relative quantity of each mRNA in the untreated small intestine samples was assumed to be an arbitrary value of 1.0. Data are represented as mean ± SEM (n = 3). (**E**) RNA was extracted from the upper small bowel (jejunum) tissues of patients suffering from Crohn’s disease (i) with (*n* = 7) and (ii) without (*n* = 13) colonic involvement. The relative quantities of GK mRNA were determined by real-time qPCR and were normalized to the quantity of β-actin mRNA. The relative quantity of each mRNA in the untreated small intestine samples was assumed to be an arbitrary value of 1.0. * indicates a statistically significant difference (Student’s *t*-test, two tailed, *P* < 0.05)

We tried to explore the mechanisms by which GK deletion in the upper small intestine augment DSS-induced colitis. Antimicrobial peptide Defensin is produced by Paneth cells that are mostly located in the upper small bowel. Quantification of the expression of defensin genes (*Defa5* and *Defa6*) in small intestine sections by quantitative PCR with reverse transcription revealed significant decreases of defensin expression in upper small bowel in *Villin-Cre;Gankyrin*^*f/f*^ mice, suggesting that GK is at least partially responsible for the transcription of defensins in this part of intestinal tract (Fig. 2a). On treatment with DSS, *Defa5* and *Defa6* mRNA expressions were significantly reduced in *Villin-Cre;Gankyrin*^*f/f*^ mice compared to that in controls (*P* < 0.05, Fig. 2b). Immunohistochemistry confirmed reduced expression of GK in the upper small bowel (jejunum) of *Villin-Cre;Gankyrin*^*f/f*^ mice, but not of *Cdx2-Cre;Gankyrin*^*f/f*^ and control mice (Fig. 2c). No difference in the number of Paneth cells was found among GK-deficient and control mice (Fig. 2c). To assess Paneth cell function, the number of granules per cell was counted. As shown in Fig. 2c, there was no difference in the number of granules. Unlike in the colon, no obvious inflammation was found both in *Villin-Cre Gankyrin*^*f/f*^ and control mice in the small intestine (Fig. 1, 2a, c, and Additional file 1: Figure S1). This might be explained by the difference in the interaction of microbiota and immune system between the small bowel and the colon [32].

To investigate another possible mechanism by which GK in the upper small bowel affects colitis, we conducted expression profiling of GK-deficient (*Villin-Cre;Gankyrin*^*f/f*^) as well as control small intestine sections using whole-genome gene expression arrays. Data analysis revealed changes in the expression of several genes related to the regulation of stem cells. We confirmed the decrease in expression of R-spondin1 and Wnt5B by quantitative RT-PCR of the GK-deficient small intestine samples relative to control samples (Fig. 2d), which was consistent with previous reports [8, 10, 33, 34]. There was no difference in the length of small bowel between *Villin-Cre Gankyrin*^*f/f*^ and control mice. At least in this model, Gankyrin deficiency does not affect intestinal regeneration that is one of stem cell functions.

Some patients with Crohn’s disease (CD) suffer from colonic involvement, while the disease is restricted to the small intestine in the remaining CD patients. Therefore, we examined whether GK in the upper small bowel contributes to colonic involvement in CD. Tissue samples of the upper small bowel from CD patients with colonic involvement showed a significantly lower level of GK expression than that in the samples from CD patients without colonic involvement (*P* < 0.05, Fig. 2e).

### GK-deficiency causes alterations in bacterial community of the gut

Host antimicrobial peptides such as defensins exhibit a major influence on the microbial community structure in gut [35]. To understand the relationship between intestinal microbiota and the augmentation of inflammation by the deficiency of GK in the small intestine, 16S rRNA gene amplicon analyses were conducted to characterize the caecum and rectum microbiota of *Villin-Cre;Gankyrin*^*f/f*^ and *Gankyrin*^*f/f*^ control mice. In total, we generated 13,442 OTUs, which contained 2,593,734 merged reads (Tables 1 and 2).

**Table 1:** Number of sequence reads derived from caecum samples

Treatment	Mice	Pairs of raw reads	Merged reads	Reads in OTUs
Untreated	GK^f/f^	45,733	37,945	19,828
		153,593	134,494	69,296
		146,664	127,381	67,536
	Villin-Cre;GK^f/f^	138,984	116,816	52,554
		205,983	173,395	81,165
		174,592	151,042	77,839
DSS-treated	GK^f/f^	111,473	89,718	67,125
		82,429	62,535	47,108
		86,110	82,334	47,630
		322,471	309,961	165,065
		256,380	245,169	142,468
	Villin-Cre;GK^f/f^	170,690	147,242	85,464
		109,655	96,143	59,270
		118,801	104,575	48,277
		215,726	185,793	101,640
		94,345	90,886	54,584
		424,553	403,679	265,611
Total		2,858,182	2,559,108	1,452,460

**Table 2:** Number of sequence reads derived from rectum samples

Treatment	Mice	Pairs of raw reads	Merged reads	Reads in OTUs
Untreated	GK^f/f^	108,219	93,088	57,378
		119,144	104,912	64,302
		132,543	116,104	56,395
	Villin-Cre;GK^f/f^	166,120	141,459	71,663
		177,145	150,617	88,569
		153,489	130,854	65,596
DSS-treated	GK^f/f^	158,088	140,514	82,872
		162,429	146,540	85,937
		146,585	129,640	72,774
		96,962	91,832	66,395
		82,258	76,967	51,798
		67,262	64,477	47,707
	Villin-Cre;GK^f/f^	146,398	129,707	76,916
		149,141	133,210	70,468
		129,604	117,091	60,096
		64,784	61,357	40,852
		58,762	55,256	41,103
		69,251	64,639	40,453
Total		2,188,184	1,948,264	1,141,274

**Fig. 3:**
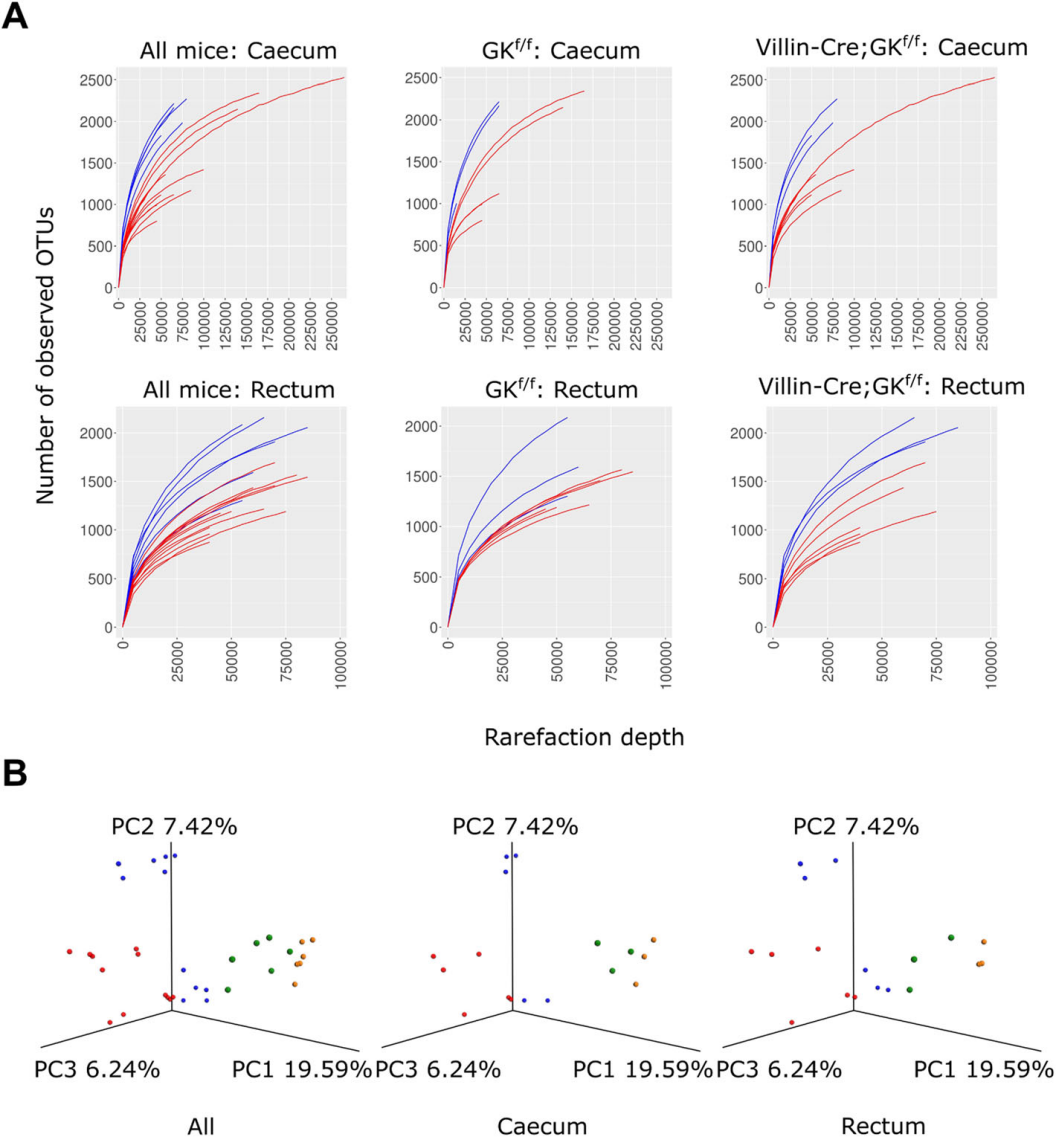
Comparison of intestinal microbiotas between samples. (**A**) The numbers of operational taxonomy units (OTUs) at different rarefaction depth are plotted for colitis non-induced mice (blue) and colitis induced mice (red). (**B**) Principal coordinate analyses of samples based on bacterial operational taxonomic unit (OTU) compositions. Compositional variations were measured by unweighted UniFrac distances. The color codes for the samples are, green: colitis non-induced *Gankyrin*^*f/f*^ control mice, orange: colitis non-induced *Villin-Cre;Gankyrin*^*f/f*^ mice, blue: colitis-induced *Gankyrin*^*f/f*^ control mice, red: colitis-induced *Villin-Cre;Gankyrin*^*f/f*^ mice

Dysbiosis is a state of microbiota which is marked by multiple factors such as a decreased bacterial community diversity and an altered bacterial composition. Dysbiosis is a frequently observed feature in the intestinal microbiota of IBD patients [36]. Consistently, colitis-induced mice showed a lower OTU richness (i.e. the number of OTUs) in comparison with their colitis non-induced counterparts in both caecum and rectum (Fig. 3a). The decreases in OTU richness of the colitis induced samples were statistically significant for both *Villin-Cre;Gankyrin*^*f/f*^ and control mice (*P* < 0.05 with Holm’s correction, Table 3). Shannon diversity index also showed a similar trend, though statistical significance was observed only between rectum samples of colitis induced and colitis non-induced *Villin-Cre;Gankyrin*^*f/f*^ mice (*P* < 0.05 with Holm’s correction, Table 3).

**Table 3:** Richness and Shannon diversity estimates of intestinal microbiota

Part	DSS	Mice	n	Observed amount of OTUs			Shannon diversity		
				Mean	Max	Min	Mean	Max	Min
Caecum	–	GK^f/f^	3	1286.0	1392.1	1124.0	6.60	6.93	6.40
	+	GK^f/f^	5	833.4*	1031.9	630.1	5.91	6.60	5.23
	–	Villin-Cre;GK^f/f^	3	1277.7	1354.4	1161.5	6.50	6.97	5.63
	+	Villin-Cre;GK^f/f^	6	830.2*	914.0	685.3	5.70	6.36	5.16
Rectum	–	GK^f/f^	3	1123.2	1412.6	905.5	6.33	6.80	5.83
	+	GK^f/f^	6	870.7*	904.8	811.9	6.04	6.36	5.66
	–	Villin-Cre;GK^f/f^	3	1261.8	1332.8	1188.6	7.09	7.99	6.52
	+	Villin-Cre;GK^f/f^	6	801.2*	1031.0	673.8	5.77*	6.31	4.63

^*^indicates a statistically significant difference (two sample *t*-test with 10,000 Monte Carlo permutations and adjustment of *P*-values by Holm’s method; *P* < 0.05)

To investigate the variation of bacterial composition among the samples, a principal coordinate analysis was conducted based on the unweighted UniFrac distances (Fig. 3b). In the plot, samples from colitis-induced mice and their colitis non-induced counterparts were clearly separated from each other. In both caecum and rectum samples, the compositional difference between these two groups were statistically significant for both *Villin-Cre;Gankyrin*^*f/f*^ and control mice (Adonis test, *P* < 0.05 with Holm’s correction, Table 4). Furthermore, many of the samples from the colitis-induced *Villin-Cre;Gankyrin*^*f/f*^ mice were placed apart from the samples of colitis-induced *Gankyrin*^*f/f*^ control mice in both caecum and rectum (Fig. 3b). The compositional difference between these two groups was statistically significant in caecum samples but not for rectum samples (Adonis test, *P* < 0.05 with Holm’s correction, Table 4). Samples from colitis non-induced *Villin-Cre;Gankyrin*^*f/f*^ mice and colitis non-induced control mice were spatially closely located in the principal coordinate plots (Fig. 3b). The two groups did not significantly differ from each other (Adonis test, *P* > 0.05) for caecum and rectum samples, though sample size was small.

**Table 4:** The corrected *P*-values and effect sizes reported by the Adonis test when performed on unweighted unifrac distances between sample groups

Part	Compared mice groups				*P*-value	Effect size
	Group A		Group B			
	DSS	Mice	DSS	Mice		
Caecum	–	GK^f/f^	–	Villin-Cre;GK^f/f^	0.1000	0.32
	+	GK^f/f^	+	Villin-Cre;GK^f/f^	0.0449*	0.15
	–	GK^f/f^	+	GK^f/f^	0.0449*	0.36
	–	Villin-Cre;GK^f/f^	+	Villin-Cre;GK^f/f^	0.0449*	0.30
Rectum	–	GK^f/f^	–	Villin-Cre;GK^f/f^	0.1782	0.32
	+	GK^f/f^	+	Villin-Cre;GK^f/f^	0.1782	0.11
	–	GK^f/f^	+	GK^f/f^	0.0478*	0.30
	–	Villin-Cre;GK^f/f^	+	Villin-Cre;GK^f/f^	0.0478*	0.29

^*^indicates a statistical significance (*P*-value is adjusted by Holm’s correction; *P* < 0.05)

We also performed a principal coordinate analysis based on weighted UniFrac distances between all samples (Additional file 2: Figure S2). In the plots, colitis-induced mice and their colitis non-induced counterparts were once again separated, though statistical significance was observed only in rectum samples of *Villin-Cre;Gankyrin*^*f/f*^ mice (Adonis test, *P* < 0.05 with Holm’s correction, Additional file 9: Table S1). A less enhanced separation between *Villin-Cre;Gankyrin*^*f/f*^ and control mice for colitis induced cases, and a clearer separation for colitis non-induced cases was observed (Additional file 2: Figure S2). Statistical significance was not observed for both cases (Adonis test, *P* > 0.05, Additional file 9: Table S1).

### Intestinal bacteria showing differential abundance in association with the GK knockout

**Fig. 4:**
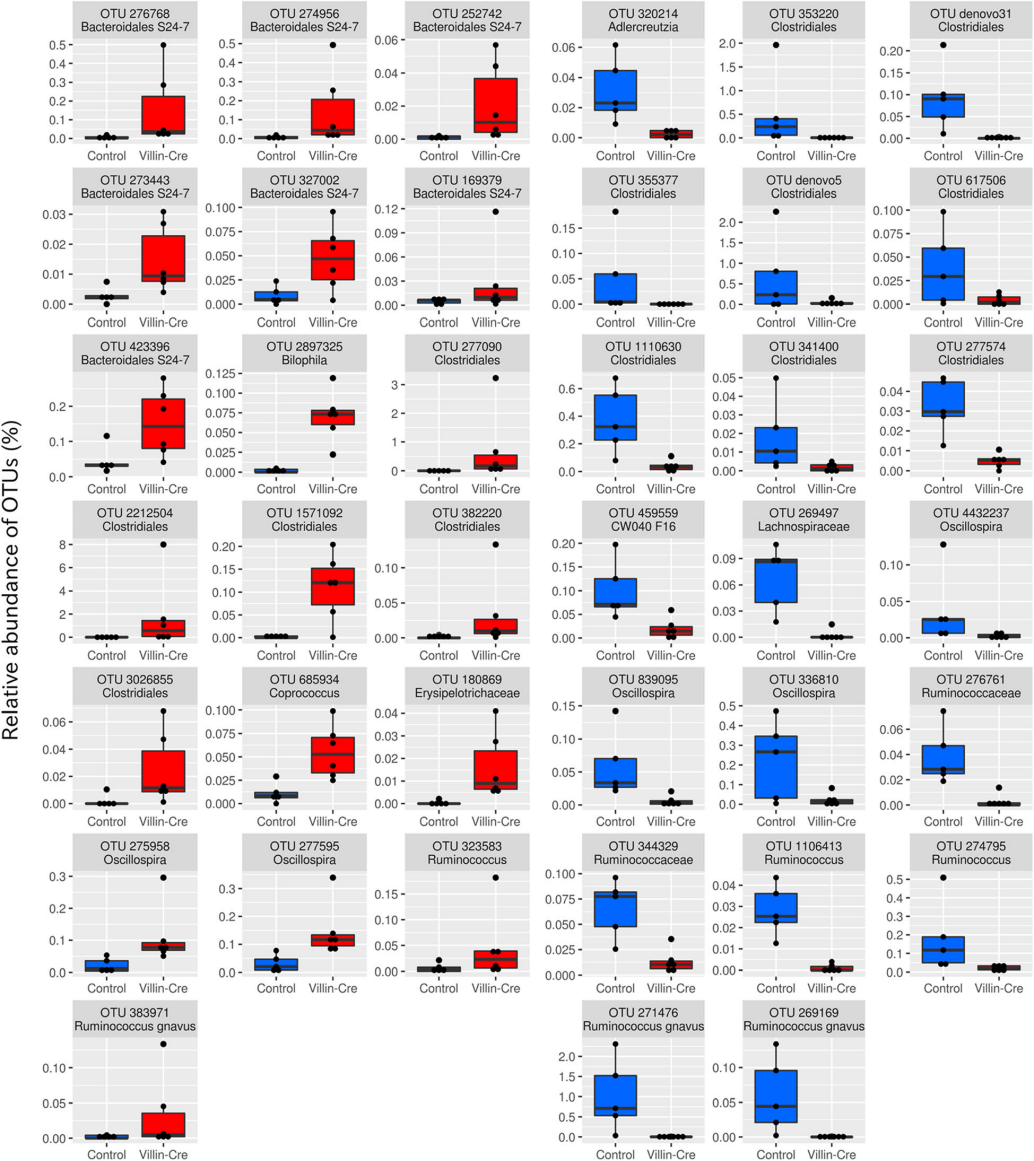
Bacterial OTUs showing differential abundance in comparison between the caecum samples of colitis-induced *Villin-Cre;Gankyrin*^*f/f*^ mice vs. colitis-induced *Gankyrin*^*f/f*^ control mice (FDR < 0.05). Each dot represents the relative abundance of OTUs within a sample

**Fig. 5:**
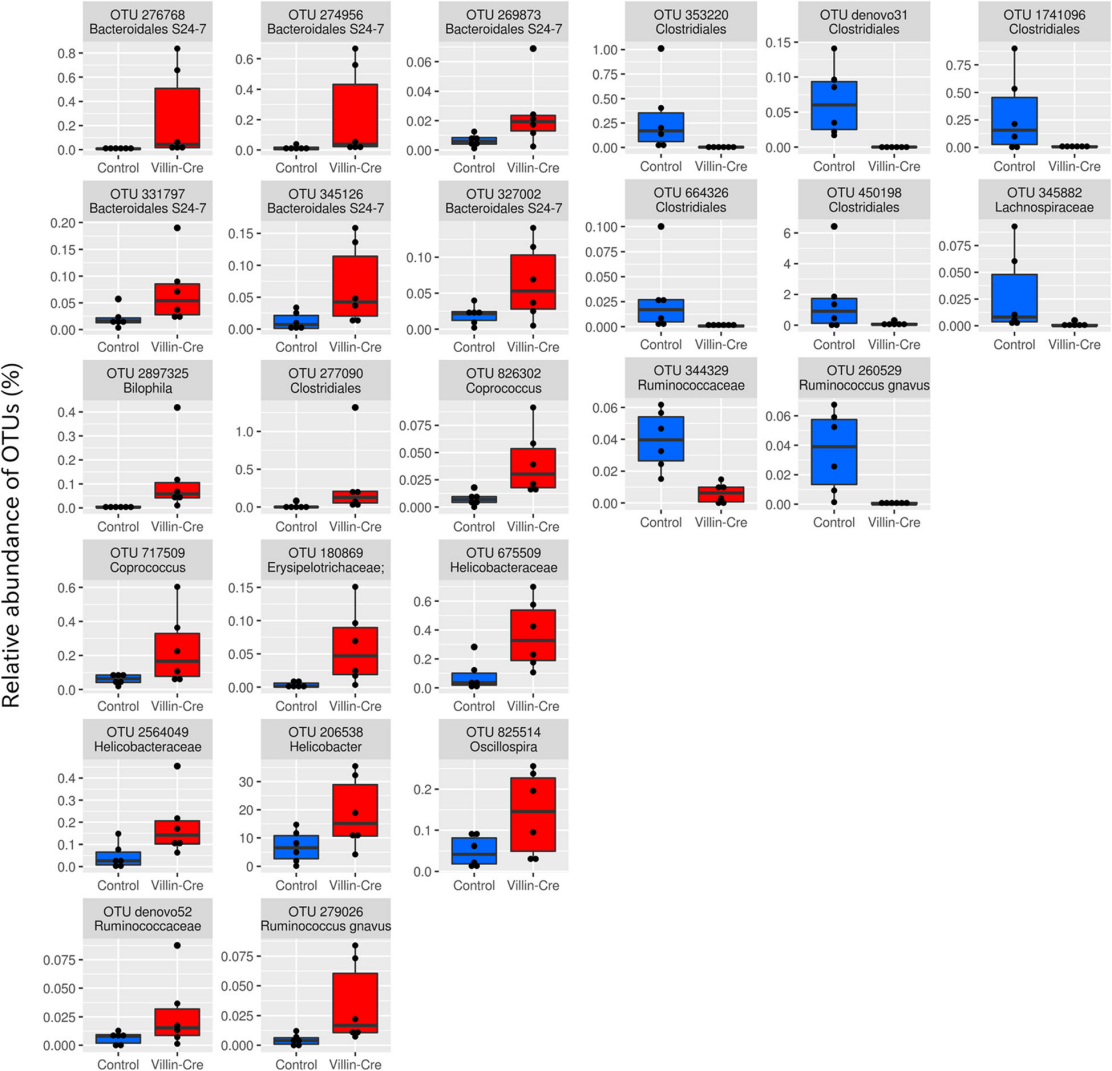
Bacterial OTUs showing differential abundance in comparison between the rectum samples of colitis-induced *Villin-Cre;Gankyrin*^*f/f*^ mice vs. colitis-induced *Gankyrin*^*f/f*^ control mice (FDR < 0.05). Each dot represents the relative abundance of OTUs within a sample

To better understand the effect of GK deficiency on the bacterial composition of intestinal microbiota, samples were compared to identify OTUs showing differential abundance. First comparisons were conducted between colitis-induced *Villin-Cre;Gankyrin*^*f/f*^ mice and colitis-induced *Gankyrin*^*f/f*^ control mice for both caecum and rectum samples. The comparisons revealed 39 OTUs in caecum and 25 OTUs in rectum with differential abundance between the groups of samples (see Methods for the criteria for differential abundance) (Figs. 4 and 5). OTUs over-represented (i.e. higher relative abundance) in *Villin-Cre;Gankyrin*^*f/f*^ mice (caecum and/or rectum samples) belonged to *Bilophila* (1 OTU, caecum and rectum), Helicobacteraceae (3 OTUs, rectum), Bacteroidales S24–7 (10 OTUs, caecum and rectum), Clostridiales (15 OTUs, caecum and rectum), and Erysipelotrichaceae (1 OTU, caecum and rectum). BLAST searches for the three OTUs belonging to Helicobacteraceae revealed that the representative sequence of OTU-206538 was aligned with the 16S rRNA gene of *Helicobacter japonicum* (NR_149210.1 and EF373968.1) with 100% sequence identify. The other two OTUs were also aligned with the 16S rRNA gene sequences of *Helicobacter* species with 100% sequence identity (GU902718.1 and AB693139.1 for OTU-675509 and OTU-2564049, respectively). OTU-206538 (*Helicobacter japonicum*) showed a notably high relative abundance (average 18.8%) in the rectum samples of *Villin-Cre;Gankyrin*^*f/f*^ mice (Fig. 5). OTUs under-represented (i.e. lower relative abundance) in caecum and/or rectum samples of colitis-induced *Villin-Cre;Gankyrin*^*f/f*^ mice belonged to *Adlercreutzia* (1 OTU, caecum), CW040 F16 (1 OTU, caecum), and Clostridiales (23 OTUs, caecum and rectum).

Next, we compared microbiotas of colitis non-induced *Villin-Cre;Gankyrin*^*f/f*^ and colitis non-induced control mice. This comparison revealed 91 OTUs in caecum and 86 OTUs in rectum with differential abundance between the two mice groups (see Methods for the criteria for differential abundance) (Additional file 3: Figure S3, Additional file 4: Figure S4, Additional file 5: Figure S5, Additional file 6: Figure S6). OTUs that were over-represented in caecum and/or rectum samples from *Villin-Cre;Gankyrin*^*f/f*^ mice belonged to Coriobacteriaceae (1 OTU, rectum), Bacteroidales (32 OTUs, caecum and rectum), *Lactobacillus reuteri* (1 OTU, rectum), *Turicibacter* (1 OTU, caecum and rectum), Clostridiales (29 OTUs, caecum and rectum), and *Allobaculum* (1 OTU, caecum and rectum). OTUs that were under-represented in caecum and/or rectum samples of *Villin-Cre;Gankyrin*^*f/f*^ mice belonged to Bacteroidales (3 OTUs, caecum and rectum), *Mucispirillum schaedleri* (1 OTU, rectum), Clostridiales (51 OTUs, caecum and rectum), Alphaproteobacteria RF32 (1 OTU, caecum), and Desulfovibrionaceae (2 OTUs, rectum).

### Elevated inflammation observed in *Villin-Cre;Gankyrin*^*f/f*^ mice is reduced through co-housing

**Fig. 6:**
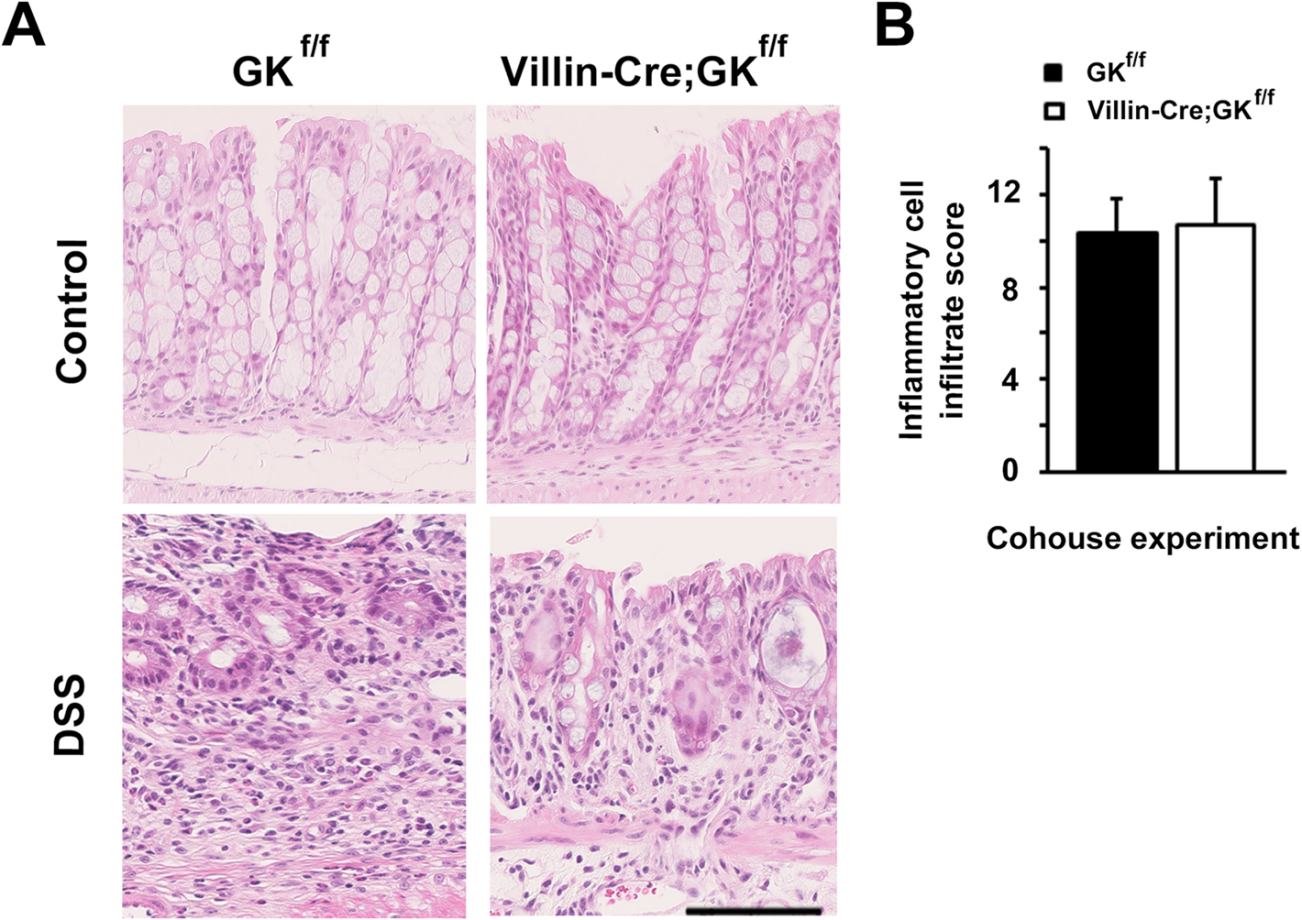
The degree of inflammatory cell infiltration was not elevated in *Villin-Cre;Gankyrin*^*f/f*^ mice when co-housed with control mice. (**A**) Representative images of hematoxylin & eosin-stained colonic tissues in co-housed *Gankyrin*^*f/f*^ (GK^f/f^) and *Villin-Cre;Gankyrin*^*f/f*^ (Villin-Cre;GK^f/f^) mice. Villin-Cre;GK^f/f^ and GK^f/f^ mice were administered with dextran sodium sulfate (DSS) in drinking water for 7 days. Scale bar, 100 μm. (**B**) Inflammatory cell infiltration into colonic tissues of co-housed GK^f/f^ and Villin-Cre;GK^f/f^ mice after 7 days of the initiation of DSS administration. n = 3 per group. * indicates a statistically significant difference (Student’s *t*-test, two tailed, *P* < 0.05)

To have insight into whether the enhanced susceptibility of *Villin-Cre;Gankyrin*^*f/f*^ mice to DSS-induced colitis is associated with intestinal microbiota, we performed a co-housing experiment between *Villin-Cre;Gankyrin*^*f/f*^ and control mice. *Villin-Cre;Gankyrin*^*f/f*^ and control mice were co-housed for 21 days before being challenged with DSS for 7 days. In this case, elevated inflammatory cell infiltration into the colon was not observed in *Villin-Cre;Gankyrin*^*f/f*^ (*P* > 0.05; Fig. 6a and b).

Microbiota analysis was also conducted by adding the 16S amplicon data from co-housed mice (Additional file 10: Table S2). Contrary to our expectation, the average OTU richness was 50% higher in *Villin-Cre;Gankyrin*^*f/f*^ mice in comparison with *Gankyrin*^*f/f*^ control mice; the average Shannon diversity index was 30% higher in *Villin-Cre;Gankyrin*^*f/f*^ mice. However, these differences were not statistically significant (*P* > 0.05, Additional file 11: Table S3). Principal component analysis based on both weighted UniFrac and unweighted UniFrac distances revealed separation of the two groups in space (Additional file 7: Figure S7), though these compositional differences were not statistically significant (Adonis test, *P* > 0.05). Comparison of the relative abundances of OTUs revealed 10 OTUs with differential abundance between the *Villin-Cre;Gankyrin*^*f/f*^ and control mice (Additional file 8: Figure S8). OTUs over-represented in *Villin-Cre;Gankyrin*^*f/f*^ mice belonged to Clostridiales (3 OTUs). OTUs under-represented in *Villin-Cre;Gankyrin*^*f/f*^ mice belonged to Helicobacteraceae (4 OTUs), Clostridiales (2 OTUs), and Enterobacteriaceae (1 OTU). The representative sequences of the four Helicobacteraceae OTUs were compared against the NCBI’s nr/nt. None of them could be assigned to *Helicobacter japonicum*.

## Discussion

Previously, we found that GK upregulates the expression of TNF-α and IL-17 in lamina propria and Kupffer cells, which was accompanied by the enhanced MAP kinase level and expansion of cancer stem cells, subsequently leading to inflammation-associated carcinogenesis [8, 10]. Regarding the mechanisms of GK-induced inflammatory responses in myeloid cells, we showed that the molecular interaction between GK and Src homology 2-containing protein tyrosine phosphatase-1 (SHP-1) leads to the activation of signal transducer and activator of transcription-3 (STAT3) and the secretion of pro-inflammatory cytokines [8, 10]. In this study, we generated two kinds of conditional GK-deficient mice: *Villin-Cre;Gankyrin*^*f/f*^ and *Cdx2-Cre;Gankyrin*^*f/f*^ mice. GK was deleted in the epithelial cells of (i) complete small intestine and colon for *Villin-Cre;Gankyrin*^*f/f*^ mice and (ii) distal small intestine and colon for *Cdx2-Cre;Gankyrin*^*f/f*^ mice. Unexpectedly, the development of colitis that was induced by DSS treatment was significantly augmented in the colon of *Villin-Cre;Gankyrin*^*f/f*^ mice as compared to that of control mice. In contrast, colitis that was induced by DSS treatment was comparable between *Cdx2-Cre;Gankyrin*^*f/f*^ and control mice. Therefore, similar to nuclear factor-κB (NF-κB) signaling [37–39], GK regulates inflammatory response in a conditional- and tissue-dependent manner.

Comparisons of gut microbiota between colitis-induced *Villin-Cre;Gankyrin*^*f/f*^ and colitis-induced control mice revealed 39 OTUs in caecum and 25 OTUs in rectum with differential abundance between the two groups of samples. Of these, two OTUs over-represented in colitis-induced *Villin-Cre;Gankyrin*^*f/f*^ mice appeared to be related to the enhanced state of inflammation: *Helicobacter japonicum* (1 OTU) and *Bilophila* spp. (1 OTU). *Helicobacter japonicum* is a recently isolated species shown to cause augmented inflammation in IL-10 deficient mice through the upregulation of the inflammatory cytokines, inducible nitric oxide synthase (iNOS), IFN-γ, IL-17A, and TNF-α [40]. Interestingly, this is consistent with the elevated expression of IFN-γ and IL-17A that we observed in colitis-induced *Villin-Cre;Gankyrin*^*f/f*^ mice, which showed augmented inflammation. In addition, two OTUs belonging to *Helicobacter* spp. were over-represented in colitis-induced *Villin-Cre;Gankyrin*^*f/f*^ mice. Enterohepatic *Helicobacter* species (EHS) are known to colonize the intestine and are also associated with IBD [41]. In fact, the elevated abundance of EHS in IBD patients in comparison to healthy subjects was revealed by a meta-analysis based on 14 clinical studies [41]. In addition, *H. pylori* known to be associated with the attenuation of IBD [42] was not detected in any of the samples examined in this study. Therefore, some of these *Helicobacter* OTUs may have contributed to the augmented inflammation in colitis-induced *Villin-Cre;Gankyrin*^*f/f*^ mice. *Bilophila* includes *B. wadsworthia,* a sulfate reducing bacterium which produces H_2_S [43]. H_2_S has been experimentally proven to promote intestinal inflammation when administered in amounts exceeding the detoxifying capacity of colonocytes in rats [44, 45]. Overall, the deficiency of GK in the small intestine during induction of colitis have led to the increased colonization of bacterial species potentially relevant to inflammation.

Between colitis non-induced *Villin-Cre;Gankyrin*^*f/f*^ mice and colitis non-induced control mice, numerous OTUs (91 OTUs in caecum and 86 OTUs in rectum) were differentially abundant. It is tempting to speculate that these differences is due to the reduced expression of defensin in the upper small bowel of *Villin-Cre;Gankyrin*^*f/f*^ mice. It is notable that none of the differentially abundant OTUs between the two groups belonged to *Helicobacter japonicum* nor *Bilophila* spp. Therefore, the distinct composition of intestinal microbiome in colitis-induced *Villin-Cre;Gankyrin*^*f/f*^ mice cannot be explained solely by the decreased defensin production. It appears that a shift in bacterial community was further induced upon colitis induction. Such an alteration of community composition may be a result of intricate dynamic interactions among intestinal inflammation, microbiome and a decreased level of defensin.

In the mice co-housing experiment, there were several OTUs with significant differential abundances. Therefore, contrary to our expectation, co-housing did not lead to similar intestinal microbiomes between *Villin-Cre;Gankyrin*^*f/f*^ and control mice. However, these differentially abundant OTUs provide some insights into the lowered inflammation for *Villin-Cre;Gankyrin*^*f/f*^ mice. There was no differentially abundant OTU related to *Helicobacter japonicum* or *Bilophila* spp. This reinforces our idea that over-representation of the OTUs related to *Helicobacter japonicum* and *Bilophila* spp. were involved in the elevated intestinal inflammation in the non-cohoused colitis-induced *Villin-Cre;Gankyrin*^*f/f*^ mice.

Epithelial antimicrobial peptides such as defensins act as important components within a complex gut barrier defense system. Deficiency of α-defensin in Paneth cells is known to cause significant changes in the composition of gut microbiota, suggesting an essential interaction between the gut microbiota and the host [46]. Our results suggest a pivotal role played by the upper small bowel in the development of colitis. Paneth cells, a major site of defensin production, are mainly located in the upper small bowel. Therefore, it can be hypothesized that lowered expression of GK by Paneth cells may affect defensin production, which in turn induces gut microbiota alteration and the development of colitis. However, whether GK is the direct regulator of α-defensin remains to be investigated. Further studies on Paneth cell-specific GK-deficient mice are necessary to test our hypothesis.

## Conclusions

The enhanced activation of GK induced by chronic inflammation promotes inflammatory response and inflammation-associated carcinogenesis [8, 10]. However, our findings illustrate the possibility of GK being involved in a negative regulation of inflammation. We propose that this negative regulatory mechanism would function by augmenting the host defense rather than by inhibiting inflammation. The augmented production of defensins and the alterations in microbiota by the small intestinal cells that express GK, may provide a compensatory mechanism that attenuates robust activation of inflammation. These results highlight the distinct site dependence of the pro- and anti-inflammatory functions of GK and provide important insights into the pathogenesis of IBD.

## Supplementary information

**Additional file 1: Figure S1.** Representative images of immunohistochemical detection of Gankyrin (GK) are shown in *Gankyrin*^*f/f*^ (GK^f/f^), *Cdx2-Cre;Gankyrin*^*f/f*^ (Cdx2-Cre;GK^f/f^) and *Villin-Cre;Gankyrin*^*f/f*^ (Villin-Cre;GK^f/f^) mice. Scale bar, 100 μm

**Additional file 2: Figure S2.** Principal coordinate analysis of weighted UniFrac distance between all samples based on bacterial operational taxonomic units (OTUs). The color codes for the samples are, green: colitis non-induced *Gankyrin*^*f/f*^ control mice, orange: colitis non-induced *Villin-Cre;Gankyrin*^*f/f*^ mice, blue: colitis-induced *Gankyrin*^*f/f*^ control mice, red: colitis-induced *Villin-Cre;Gankyrin*^*f/f*^ mice.

**Additional file 3: Figure S3.** Bacterial OTUs under-represented in caecum samples of colitis non-induced *Villin-Cre;Gankyrin*^*f/f*^ mice in comparison with caecum samples of colitis non-induced *Gankyrin*^*f/f*^ control mice (FDR < 0.05). Each dot represents the relative abundance of OTUs within each sample.

**Additional file 4: Figure S4.** Bacterial OTUs over-represented in caecum samples of colitis non-induced *Villin-Cre;Gankyrin*^*f/f*^ mice in comparison with caecum samples of colitis non-induced *Gankyrin*^*f/f*^ control mice (FDR < 0.05). Each dot represents the relative abundance of OTUs within each sample.

**Additional file 5: Figure S5.** Bacterial OTUs under-represented in rectum samples of colitis non-induced *Villin-Cre;Gankyrin*^*f/f*^ mice in comparison with rectum samples of colitis non-induced *Gankyrin*^*f/f*^ control mice (FDR < 0.05). Each dot represents the relative abundance of OTUs within each sample.

**Additional file 6: Figure S6.** Bacterial OTUs over-represented in rectum samples of colitis non-induced *Villin-Cre;Gankyrin*^*f/f*^ mice in comparison with rectum samples of colitis non-induced *Gankyrin*^*f/f*^ control mice (FDR < 0.05). Each dot represents the relative abundance of OTUs within each sample.

**Additional file 7: Figure S7.** Principal coordinate analysis of (A) unweighted UniFrac distance and (B) weighted UniFrac distance between all samples, including samples from cohouse mice, based on bacterial operational taxonomic units (OTUs). The color codes for the samples are, light blue: co-housed colitis-induced *Gankyrin*^*f/f*^ control mice, pink: co-housed colitis-induced *Villin-Cre;Gankyrin*^*f/f*^ mice, green: colitis non-induced Gankyrin^f/f^ control mice, orange: colitis non-induced Villin-Cre;Gankyrin^f/f^ mice, blue: colitis-induced *Gankyrin*^*f/f*^ control mice, red: colitis-induced *Villin-Cre;Gankyrin*^*f/f*^ mice.

**Additional file 8: Figure S8.** Bacterial OTUs showing differential abundance in comparison between the rectum samples of co-housed colitis-induced *Villin-Cre;Gankyrin*^*f/f*^ mice vs. co-housed colitis-induced *Gankyrin*^*f/f*^ control mice (FDR < 0.05). Each dot represents the relative abundance of OTUs within each sample.

**Additional file 9: Table S1.** The corrected *P*-values and effect sizes reported by the Adonis test when performed on weighted unifrac distances between sample groups

**Additional file 10: Table S2.** Number of sequence reads analyzed during the 16S rRNA amplicon analysis conducted to understand the intestinal microbiota in the co-housed mice groups

**Additional file 11: Table S3.** Richness and Shannon diversity estimates of intestinal microbiota of co-housed *Gankyrin*^*f/f*^ control mice and co-housed colitis induced *Villin-Cre;Gankyrin*^*f/f*^ mice

## Data Availability

Sequence data of the raw paired-end reads were deposited to DNA Data Bank of Japan/Sequence Read Archive (DDBJ/DRA) and are accessible through the accession number DRA009279. The merged reads can be obtained from the following site: ftp://ftp.genome.jp/pub/db/community/microbiome_kindai/.
