# Supplementary figures and images for: Deficiency of Gankyrin in the small intestine is associated with augmented colitis accompanied by altered bacterial composition of intestinal microbiota
Source: BMC Gastroenterol. 2020 Jan 15;20:12. doi: 10.1186/s12876-019-1156-0 (PMC6964040; doi:10.1186/s12876-019-1156-0)

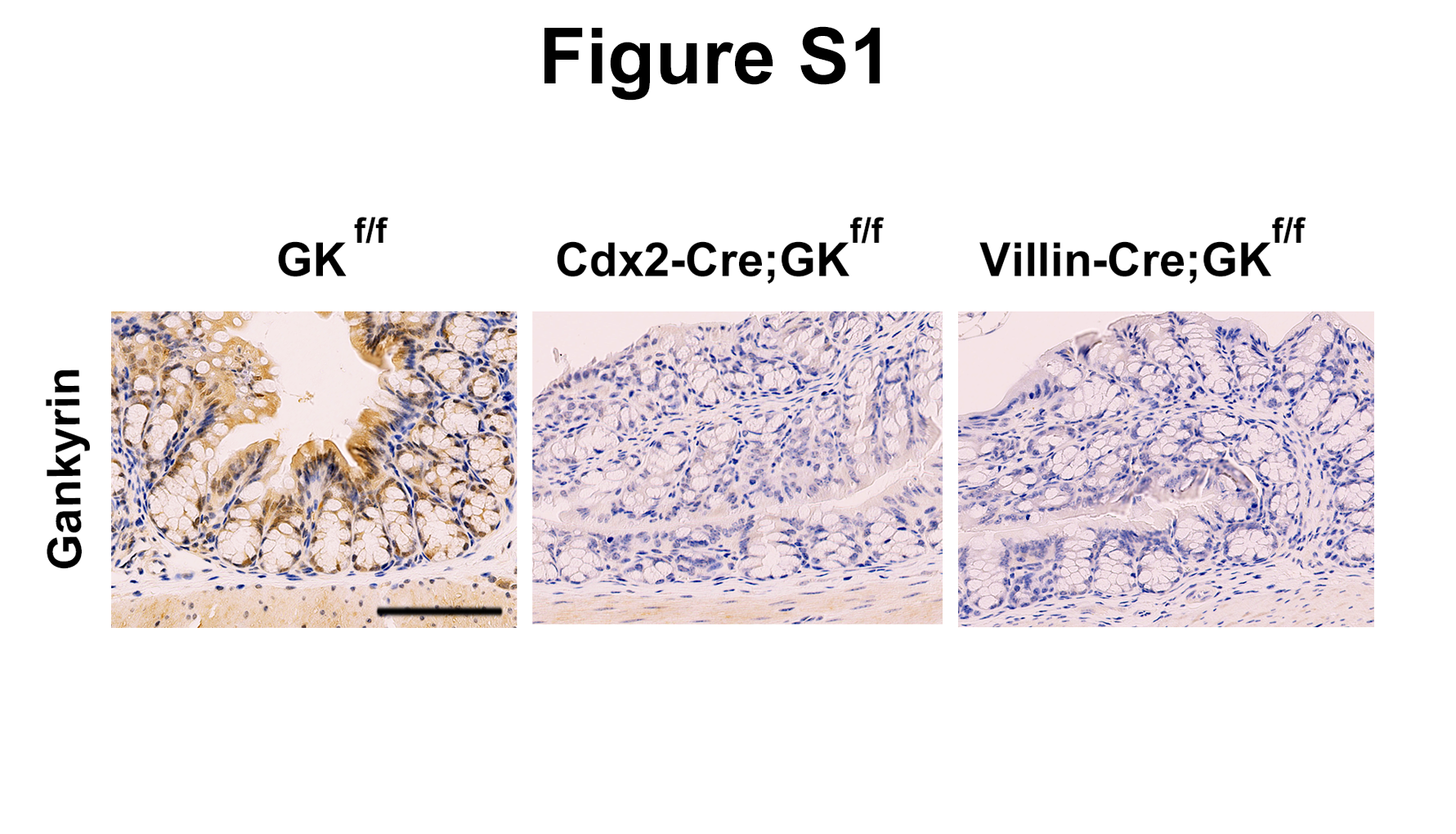

Supplement: Supplementary file 1 — Additional file 1: Figure S1. Representative images of immunohistochemical detection of Gankyrin (GK) are shown in Gankyrinf/f (GKf/f), Cdx2-Cre;Gankyrinf/f (Cdx2-Cre;GKf/f) and Villin-Cre;Gankyrinf/f (Villin-Cre;GKf/f) mice. Scale bar, 100 μm [file 12876_2019_1156_MOESM1_ESM.tif]

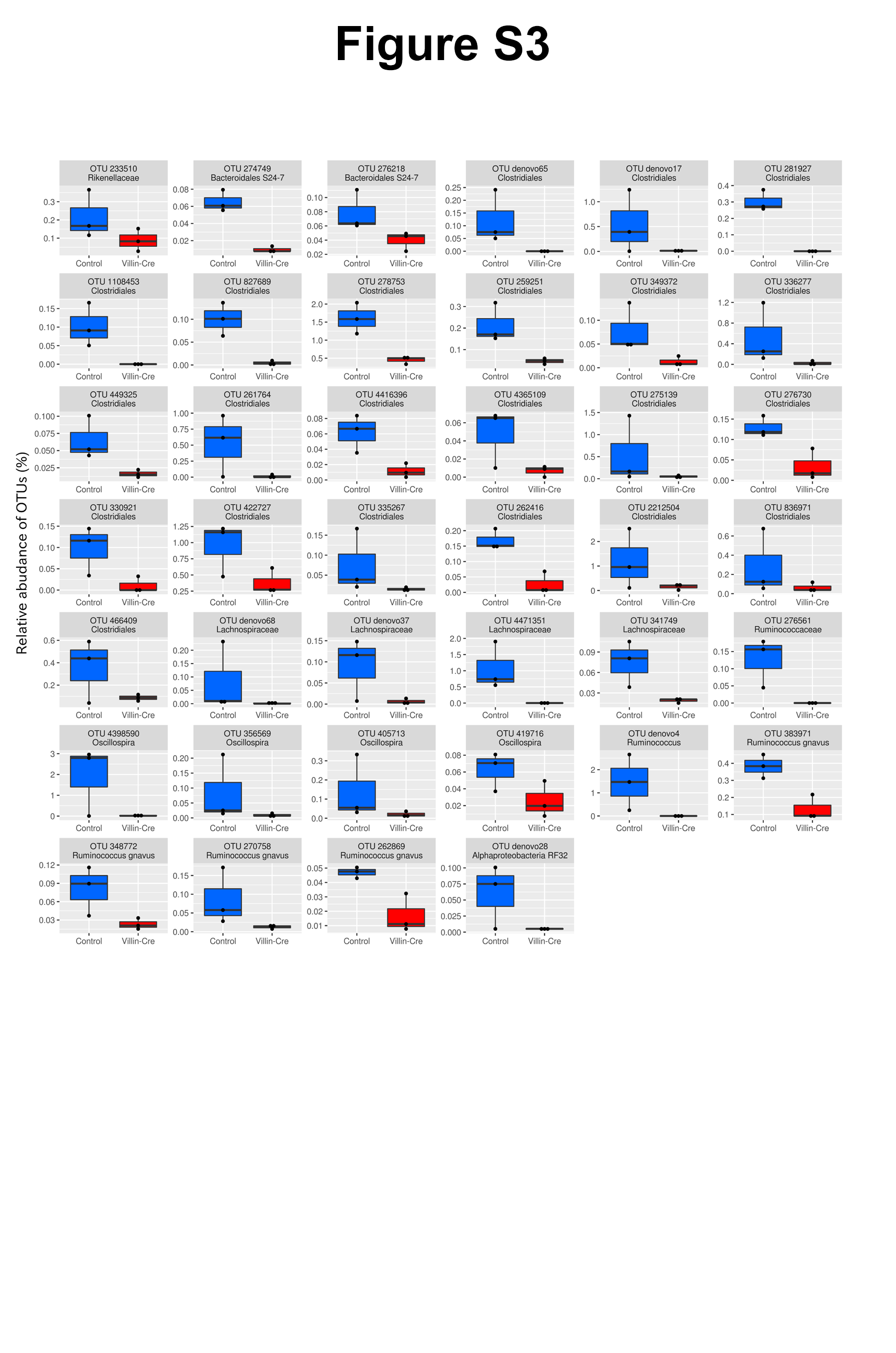

Supplement: Supplementary file 3 — Additional file 3: Figure S3. Bacterial OTUs under-represented in caecum samples of colitis non-induced Villin-Cre;Gankyrinf/f mice in comparison with caecum samples of colitis non-induced Gankyrinf/f control mice (FDR < 0.05). Each dot represents the relative abundance of OTUs within each sample. [file 12876_2019_1156_MOESM3_ESM.tif]

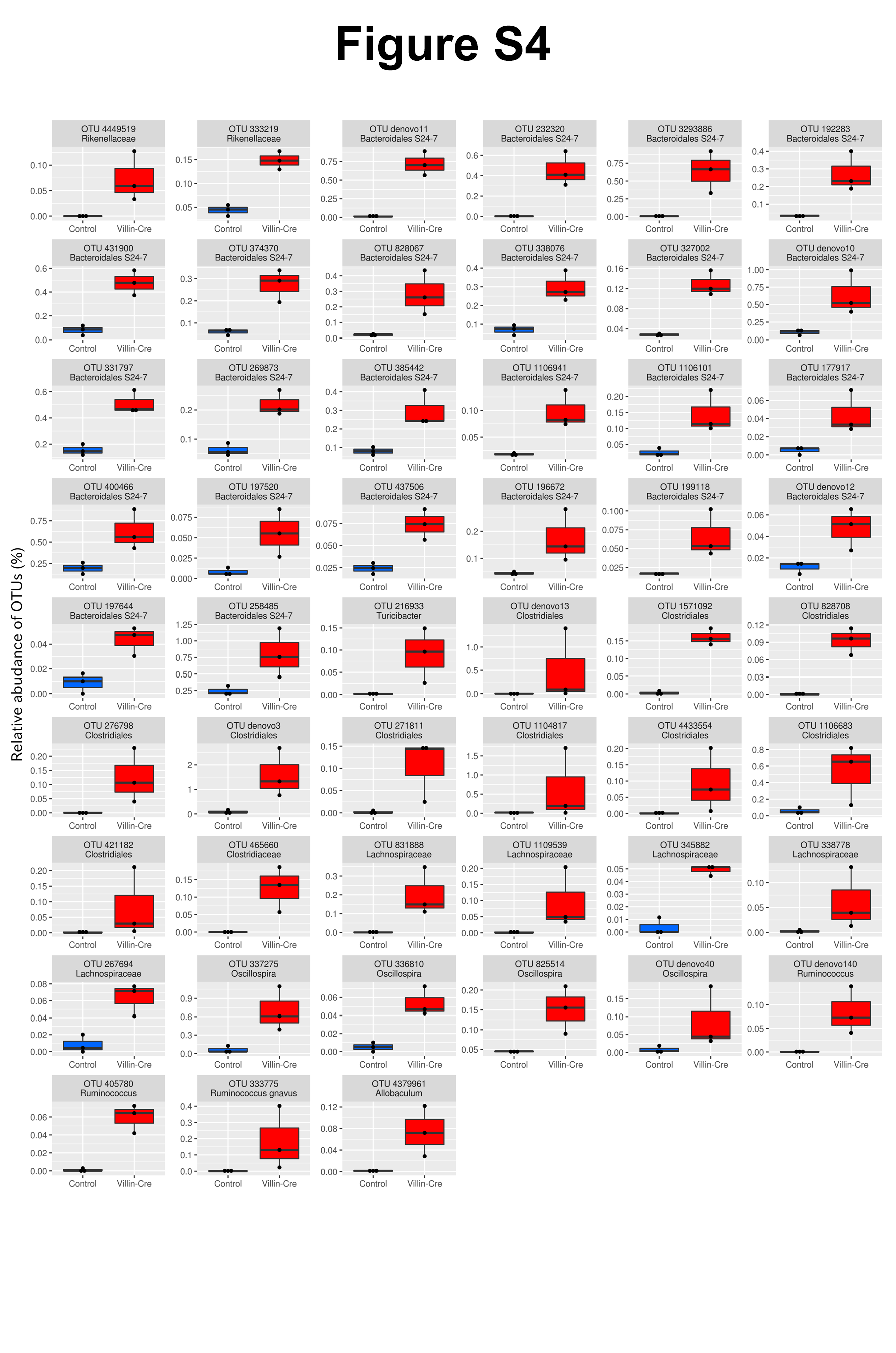

Supplement: Supplementary file 4 — Additional file 4: Figure S4. Bacterial OTUs over-represented in caecum samples of colitis non-induced Villin-Cre;Gankyrinf/f mice in comparison with caecum samples of colitis non-induced Gankyrinf/f control mice (FDR < 0.05). Each dot represents the relative abundance of OTUs within each sample. [file 12876_2019_1156_MOESM4_ESM.tif]

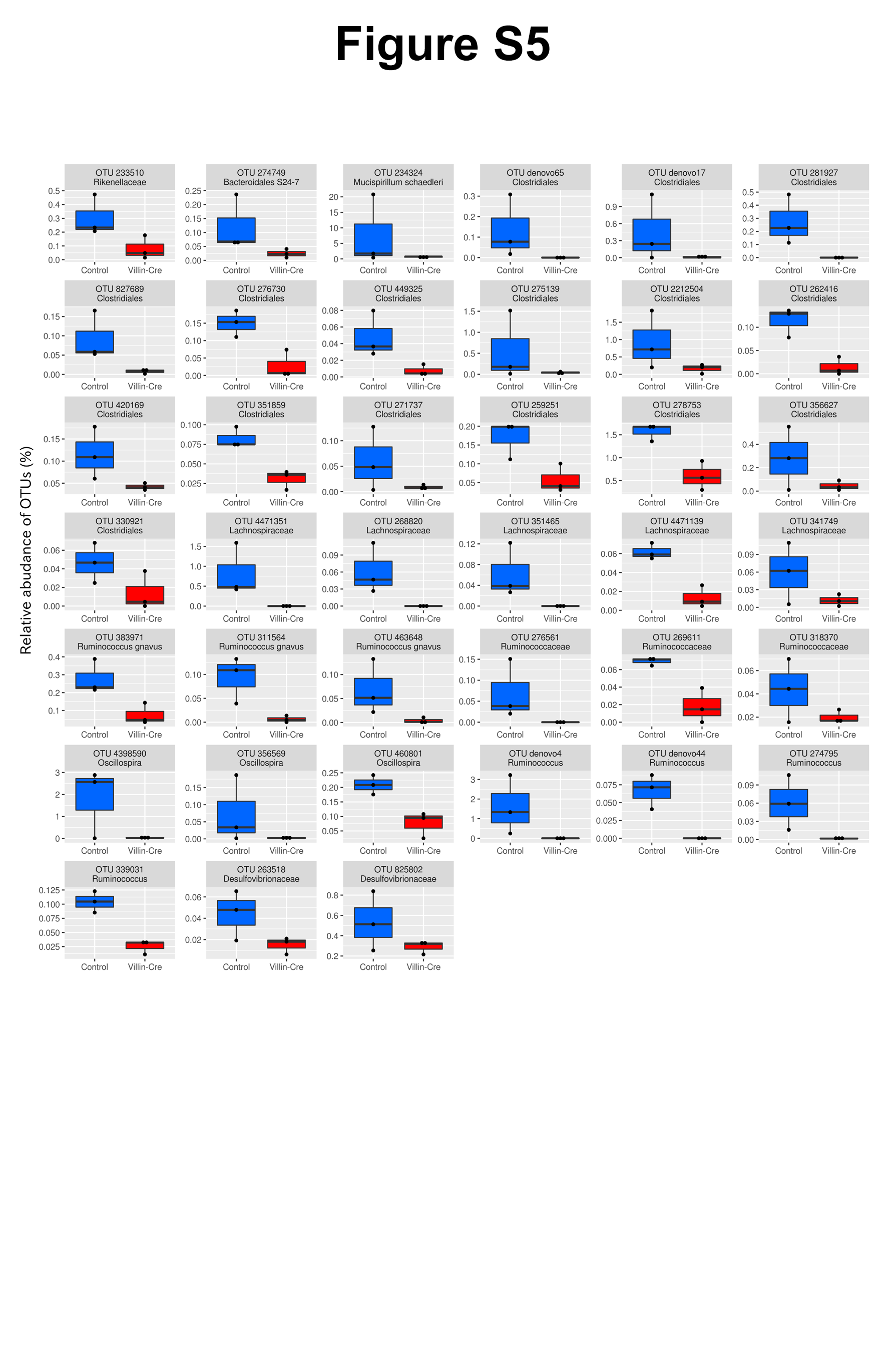

Supplement: Supplementary file 5 — Additional file 5: Figure S5. Bacterial OTUs under-represented in rectum samples of colitis non-induced Villin-Cre;Gankyrinf/f mice in comparison with rectum samples of colitis non-induced Gankyrinf/f control mice (FDR < 0.05). Each dot represents the relative abundance of OTUs within each sample. [file 12876_2019_1156_MOESM5_ESM.tif]

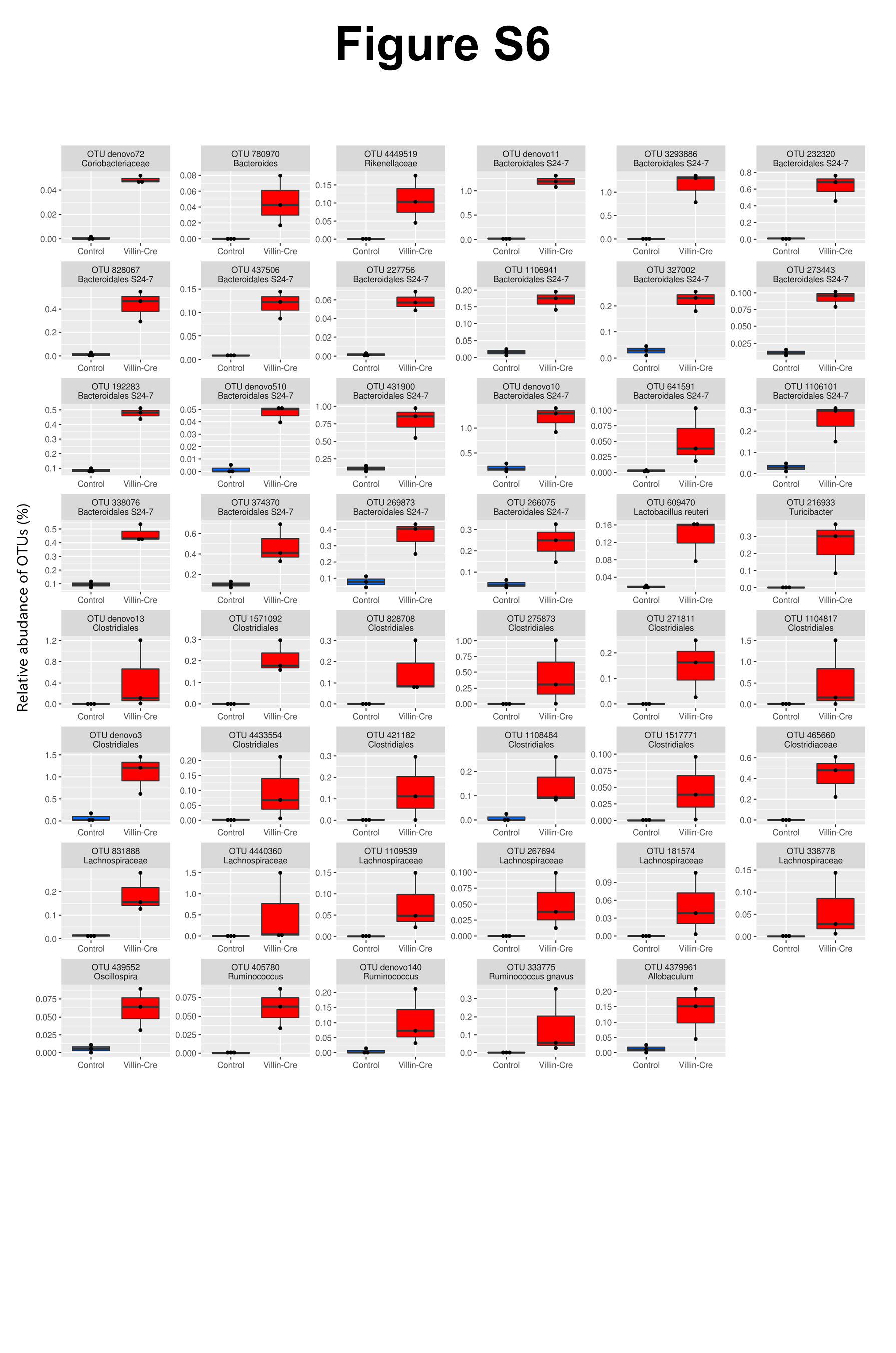

Supplement: Supplementary file 6 — Additional file 6: Figure S6. Bacterial OTUs over-represented in rectum samples of colitis non-induced Villin-Cre;Gankyrinf/f mice in comparison with rectum samples of colitis non-induced Gankyrinf/f control mice (FDR < 0.05). Each dot represents the relative abundance of OTUs within each sample. [file 12876_2019_1156_MOESM6_ESM.tif]

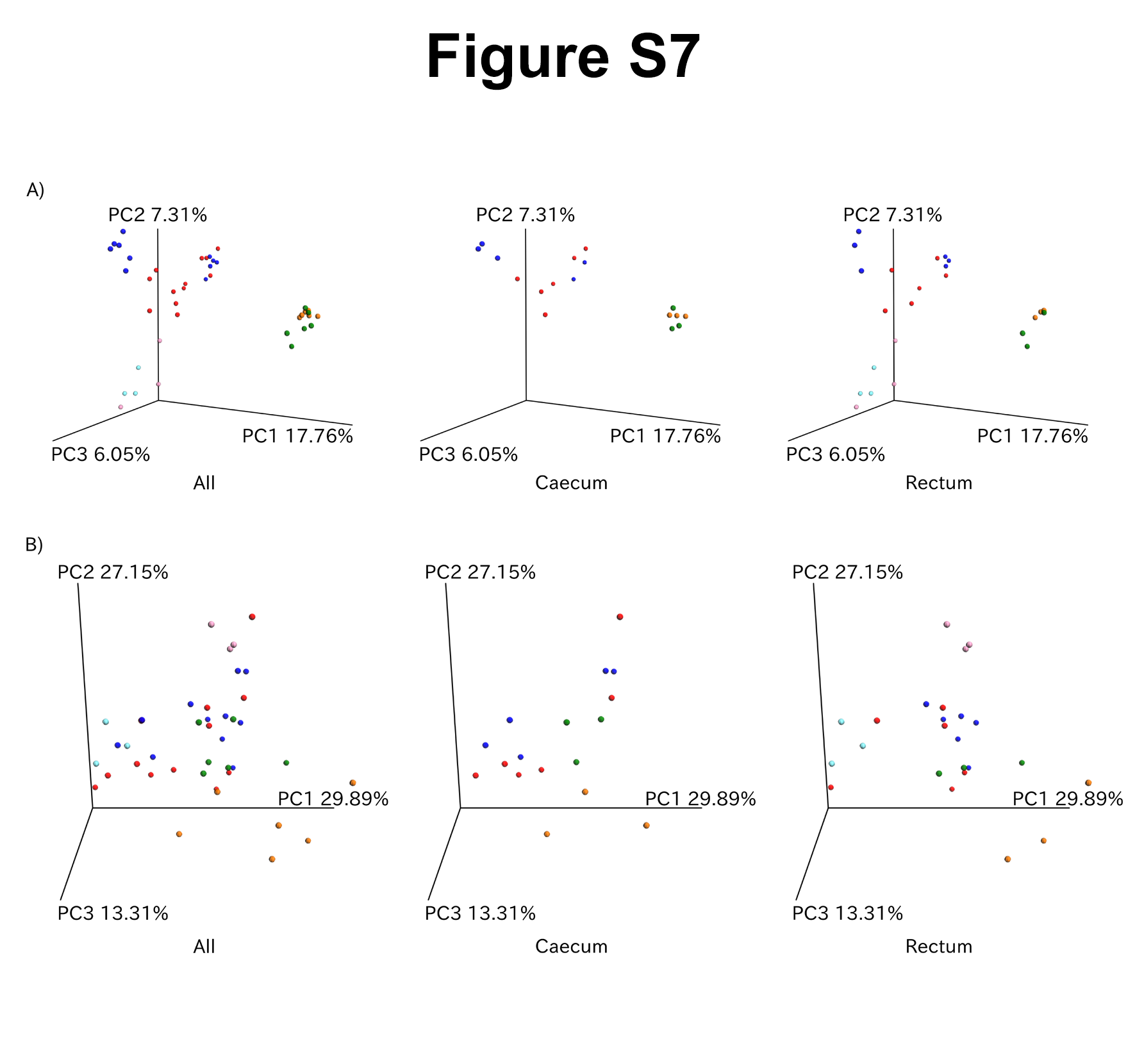

Supplement: Supplementary file 7 — Additional file 7: Figure S7. Principal coordinate analysis of (A) unweighted UniFrac distance and (B) weighted UniFrac distance between all samples, including samples from cohouse mice, based on bacterial operational taxonomic units (OTUs). The color codes for the samples are, light blue: co-housed colitis-induced Gankyrinf/f control mice, pink: co-housed colitis-induced Villin-Cre;Gankyrinf/f mice, green: colitis non-induced Gankyrinf/f control mice, orange: colitis non-induced Villin-Cre;Gankyrinf/f mice, blue: colitis-induced Gankyrinf/f control mice, red: colitis-induced Villin-Cre;Gankyrinf/f mice. [file 12876_2019_1156_MOESM7_ESM.tif]

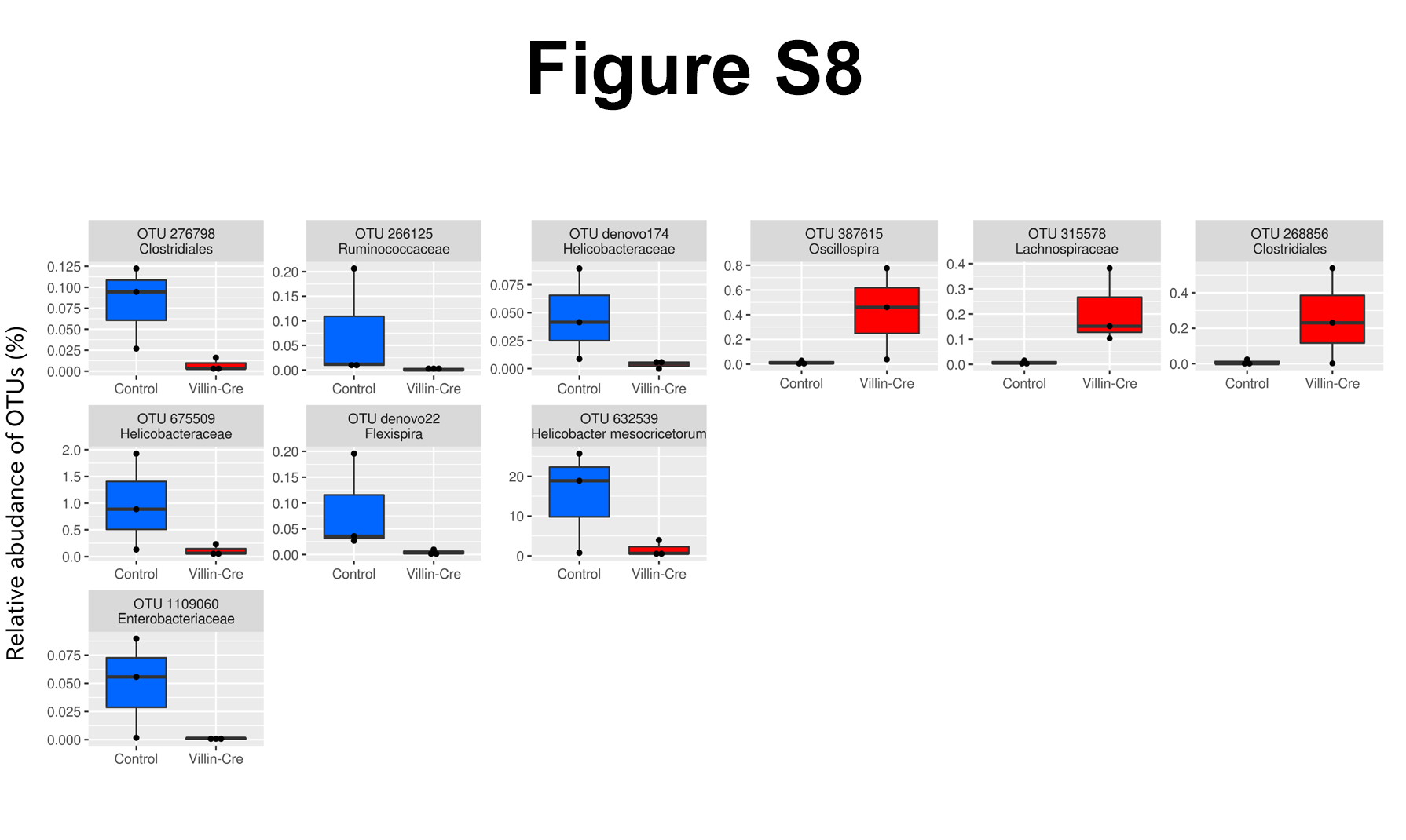

Supplement: Supplementary file 8 — Additional file 8: Figure S8. Bacterial OTUs showing differential abundance in comparison between the rectum samples of co-housed colitis-induced Villin-Cre;Gankyrinf/f mice vs. co-housed colitis-induced Gankyrinf/f control mice (FDR < 0.05). Each dot represents the relative abundance of OTUs within each sample. [file 12876_2019_1156_MOESM8_ESM.tif]
