# Supplementary material for: Deficiency of Gankyrin in the small intestine is associated with augmented colitis accompanied by altered bacterial composition of intestinal microbiota
Source: BMC Gastroenterol. 2020 Jan 15;20:12. doi: 10.1186/s12876-019-1156-0 (PMC6964040; doi:10.1186/s12876-019-1156-0)
Supplement: Supplementary file 9 — Additional file 9: Table S1. The corrected P-values and effect sizes reported by the Adonis test when performed on weighted unifrac distances between sample groups [file 12876_2019_1156_MOESM9_ESM.docx]

Table S1. The corrected *P*-values and effect sizes reported by the Adonis test when performed on weighted unifrac distances between sample groups

| Part | Compared mice groups | | | | *P*-value | Effect size |
| --- | --- | --- | --- | --- | --- | --- |
|  | Group A | | Group B | |  |  |
|  | DSS | Mice | DSS | Mice |  |  |
| Caecum | - | GK^f/f^ | - | Villin-Cre;GK^f/f^ | 0.3820 | 0.50 |
|  | + | GK^f/f^ | + | Villin-Cre;GK^f/f^ | 0.6136 | 0.06 |
|  | - | GK^f/f^ | + | GK^f/f^ | 0.5367 | 0.20 |
|  | - | Villin-Cre;GK^f/f^ | + | Villin-Cre;GK^f/f^ | 0.3820 | 0.24 |
| Rectum | - | GK^f/f^ | - | Villin-Cre;GK^f/f^ | 0.2000 | 0.56 |
|  | + | GK^f/f^ | + | Villin-Cre;GK^f/f^ | 0.2343 | 0.12 |
|  | - | GK^f/f^ | + | GK^f/f^ | 0.1204 | 0.27 |
|  | - | Villin-Cre; GK^f/f^ | + | Villin-Cre;GK^f/f^ | 0.0497* | 0.48 |

* indicates a statistical significance (*P*-value is adjusted by Holm’s correction; *P*<0.05)
