# Supplementary material for: Deficiency of Gankyrin in the small intestine is associated with augmented colitis accompanied by altered bacterial composition of intestinal microbiota
Source: BMC Gastroenterol. 2020 Jan 15;20:12. doi: 10.1186/s12876-019-1156-0 (PMC6964040; doi:10.1186/s12876-019-1156-0)
Supplement: Supplementary file 11 — Additional file 11: Table S3. Richness and Shannon diversity estimates of intestinal microbiota of co-housed Gankyrinf/f control mice and co-housed colitis induced Villin-Cre;Gankyrinf/f mice [file 12876_2019_1156_MOESM11_ESM.docx]

Table S3. Richness and Shannon diversity estimates of intestinal microbiota of co-housed *Gankyrin^f/f^* control mice and co-housed colitis induced *Villin-Cre;Gankyrin^f/f^* mice

| Mice | n | Observed amount of OTUs | | | Shannon diversity | | |
| --- | --- | --- | --- | --- | --- | --- | --- |
|  |  | Mean | Max | Min | Mean | Max | Min |
| GK^f/f^ | 3 | 611.2 | 674.0 | 567.2 | 4.74 | 5.45 | 4.07 |
| Villin-Cre;GK^f/f^ | 3 | 916.5 | 998.9 | 875.1 | 6.17 | 7.04 | 5.33 |

No statistical significance was observed for the above pair for both measure (two sample *t*-test with 10,000 Monte Carlo permutations; *P*>0.05)
